# Raman Spectroscopy-Based Measurements of Single-Cell Phenotypic Diversity in Microbial Populations

**DOI:** 10.1128/mSphere.00806-20

**Published:** 2020-10-28

**Authors:** Cristina García-Timermans, Ruben Props, Boris Zacchetti, Myrsini Sakarika, Frank Delvigne, Nico Boon

**Affiliations:** a Center for Microbial Ecology and Technology (CMET), Faculty of Bioscience Engineering, Ghent University, Ghent, Belgium; b TERRA Research and Teaching Centre, Microbial Processes and Interactions (MiPI), Université de Liège—Gembloux Agro-Bio Tech, Gembloux, Belgium; National Institute of Advanced Industrial Science and Technology

**Keywords:** Raman spectroscopy, microbial population, stress, phenotypic diversity, single-cell analysis, Hill numbers, *Escherichia coli*, *Saccharomyces cerevisiae*

## Abstract

Microbial cells experience physiological changes due to environmental change, such as pH and temperature, the release of bactericidal agents, or nutrient limitation. This has been shown to affect community assembly and physiological processes (e.g., stress tolerance, virulence, or cellular metabolic activity). Metabolic stress is typically quantified by measuring community phenotypic properties such as biomass growth, reactive oxygen species, or cell permeability. However, bulk community measurements do not take into account single-cell phenotypic diversity, which is important for a better understanding and the subsequent management of microbial populations. Raman spectroscopy is a nondestructive alternative that provides detailed information on the biochemical makeup of each individual cell. Here, we introduce a method for describing single-cell phenotypic diversity using the Hill diversity framework of Raman spectra. Using the biomolecular profile of individual cells, we obtained a metric to compare cellular states and used it to study stress-induced changes. First, in two Escherichia coli populations either treated with ethanol or nontreated and then in two Saccharomyces cerevisiae subpopulations with either high or low expression of a stress reporter. In both cases, we were able to quantify single-cell phenotypic diversity and to discriminate metabolically stressed cells using a clustering algorithm. We also described how the lipid, protein, and nucleic acid compositions changed after the exposure to the stressor using information from the Raman spectra. Our results show that Raman spectroscopy delivers the necessary resolution to quantify phenotypic diversity within individual cells and that this information can be used to study stress-driven metabolic diversity in microbial populations.

**IMPORTANCE** Microbial cells that live in the same community can exist in different physiological and morphological states that change as a function of spatiotemporal variations in environmental conditions. This phenomenon is commonly known as phenotypic heterogeneity and/or diversity. Measuring this plethora of cellular expressions is needed to better understand and manage microbial processes. However, most tools to study phenotypic diversity only average the behavior of the sampled community. In this work, we present a way to quantify the phenotypic diversity of microbial samples by inferring the (bio)molecular profile of its constituent cells using Raman spectroscopy. We demonstrate how this tool can be used to quantify the phenotypic diversity that arises after the exposure of microbes to stress. Raman spectroscopy holds potential for the detection of stressed cells in bioproduction.

## Abstract (precis)

Microbial cells that live in the same community can exist in different physiological and morphological states that change as a function of spatiotemporal variations in environmental conditions. This phenomenon is commonly known as phenotypic heterogeneity and/or diversity. Measuring this plethora of cellular expressions is needed to better understand and manage microbial processes. However, most tools to study phenotypic diversity only average the behavior of the sampled community. In this work, we present a way to quantify the phenotypic diversity of microbial samples by inferring the (bio)molecular profile of its constituent cells using Raman spectroscopy. We demonstrate how this tool can be used to quantify the phenotypic diversity that arises after the exposure of microbes to stress. Raman spectroscopy holds potential for the detection of stressed cells in bioproduction.

## INTRODUCTION

Monoclonal microbial populations can exhibit heterogeneous genetic expression, which underlies phenotypic differences between cells. Phenotypic diversity has been shown to increase population survival or fitness in a changing environment and allows microorganisms to divide tasks and organize as a group. This differential gene expression can arise due to environmental pressure, stochastic events, periodic oscillations, or cell-to-cell interactions (1–3). When a deviation from optimal growth conditions occurs, such as changes in temperature, pH, nutrients, salts, and/or oxygen levels, a stress response is triggered in microorganisms (both prokaryotes and eukaryotes), resulting in a biochemical cascade to promote stress tolerance, virulence, or other physiological changes. These strategies can result in enhanced survival, virulence, cross-protection, or cell death (4–6). Usually, microorganisms show mixed behavioral strategies, maximizing the chances of survival (7), making phenotypic diversity a crucial characteristic of stress-driven phenotypes. However, cellular stress is often measured at the community level using bulk technologies, such as cell concentration, quantity of reactive oxygen species (ROS), cell permeability, or protein content. While these methods reveal important information, they provide average information for the whole population, thereby failing to describe cell-to-cell variability and bet-hedging strategies (8). To better understand stress-driven changes, single-cell technologies provide new opportunities.

There are several single-cell technologies available to study the response of individual cells to stress. For example, fluorescent labels that tag certain cellular functions (membrane potential, intracellular enzyme activity, or a stress reporter) can be used in combination with flow cytometry (9, 10) or imaging techniques (11). Single-cell (multi)omics opens the door to a very detailed understanding of the metabolism of individual cells, although it is a low-throughput technique that still presents many challenges in its accuracy (12). Raman spectroscopy is an alternative single-cell tool that can detect individual phenotypes without the use of fluorescent probes. It is an optical method that uses a laser to excite the molecules present in the cell and records their inelastic scattering, thereby generating a single-cell fingerprint that contains (semi)quantitative information on its constituent molecules, such as nucleic acids, proteins, lipids, and carbohydrates. This technique has been used to study stress-induced phenotypic differences of the cyanobacterium *Synechocystis* sp. (13): the fingerprints of cells treated with different concentrations of acetate or NaCl and nontreated cells were differentiable using the discriminant analysis of principal-component analysis (PCA). Also, Teng and colleagues (14) found that Escherichia coli cells exposed to several antibiotics, alcohols, and chemicals had distinct Raman fingerprints. However, there are currently no quantitative methods to describe phenotypic diversity in single cells using their unlabeled Raman spectra.

A widely used set of metrics to quantify the diversity of microbial communities are Hill numbers, also known as the effective number of species, as they express in intuitive units the number of equally abundant species that are needed to match the value of the Hill number. Hill numbers respect other important ecological principles, such as the replication principle, which states that in a group with *N* equally diverse groups that have no species in common, the diversity of the pooled groups must be *N* times the diversity of a single group (15, 16). They are commonly used to quantify microbial diversity based on 16S rRNA sequencing techniques but have also been applied to flow cytometry yielding similar results (17). However, phenotypic diversity at the single-cell level—defined as the diversity of observable characteristics or traits in single cells—has not yet been described. This would require multiparametric information of individual cells, something Raman spectroscopy can provide.

Quantifying phenotypic diversity at the single-cell level could be useful to follow and manage stress in bioproduction: to maintain high bioproduction rates, it is important to find or create stress-tolerant organisms. For instance, in microbial production of alcohol (considered a sustainable alternative source for chemicals and fuels), one of the major limitations is the toxicity and/or growth inhibition caused by the alcohol that is produced. The alcohol increases the fluidity of the cell membrane and causes a disruption of the phospholipid components that inhibits growth and can lead to death. It also affects nutrient uptake and ion transport. Therefore, there have been efforts in evolutionary and synthetic engineering to increase alcohol tolerance in several organisms, for example, Escherichia coli and Saccharomyces cerevisiae, widely used in bioproduction (18).

Here, we describe a method to quantify single-cell phenotypic diversity using Raman spectroscopy based on the Hill diversity framework. We defined the necessary steps to preprocess Raman spectra and demonstrated its integration into the Hill diversity framework. The necessary functionalities were also embedded in the open source MicroRaman package (https://github.com/CMET-UGent/MicroRaman). To illustrate the use of this method, we applied it to two popular strains in bioproduction. First, we compared an E. coli population under stress conditions (cultivated with ethanol) with a control population. Second, we applied the method to two subpopulations of a green fluorescent protein (GFP)-labeled S. cerevisiae culture that was grown under nutrient-limiting conditions. In both cases, we show how the stress-induced single-cell phenotypic diversity can be quantified using the Raman spectra of the single cells and how this information can be used to detect a shift in the phenotype of the population. Finally, we use this information to infer how the molecular profile of the cells changes after being exposed to the stressors.

## RESULTS

### Phenotypic diversity quantification of Raman spectra using Hill numbers.

**FIG 1:**
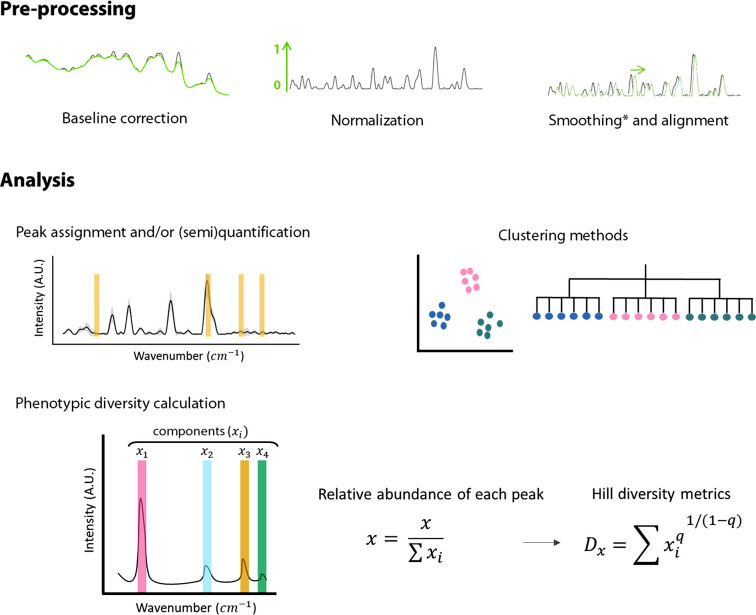
Summary of the preprocessing and analysis of the Raman spectra. First, the baseline is corrected and the spectra are normalized. Spectra can be smoothed and aligned; however, smoothing can erase potentially relevant information and should be carefully considered. Similarly, alignment can produce faulty spectra by displacing the signal and thus need to be used reasonably. Once the spectra are preprocessed, it is possible to (i) extract (semi)quantitative information, (ii) cluster cells or create phenotypic trees, or (iii) calculate the single-cell phenotypic diversity. For the latter, Raman peaks that correspond to one or several metabolites are considered components. The intensity of these components (*x*) is used to quantify phenotypic diversity. The number of components is *i*. The order of diversity (*q*) can be 0, 1, or 2, meaning that richness, evenness, or both parameters, respectively, are considered in the metric. This equation considers richness and estimated evenness of metabolites in a single cell.

To infer the phenotypic properties of individual cells, raw Raman spectra data need to be subjected to quality control and trimming steps (Fig. 1, preprocessing). This step aims to remove noise from spectra to be able to extract meaningful biological information. First, the spectrum that contains cosmic rays needs to be removed manually or automatically (19). Then, we select the spectral region that is most relevant for microbial fingerprinting, around 500 to 2,000 cm^−1^ (20). Once this region of the spectrum is selected, the first step in the preprocessing is to correct the baseline, which can be degraded due to instrument fluctuations or background signal influence (19, 21). Then, the spectra are normalized to avoid the absolute intensity from masking the variation of signals of interest (22, 23). It is also possible to align and/or smooth the Raman signal, but these steps can introduce noise to the measurements or remove relevant information and thus should be carefully considered.

After the spectra have been preprocessed, several analyses can be performed (Fig. 1, analysis). For example, peaks of interest can be selected for semiquantitative analysis or quantitative analysis using a calibration curve (24). Also, the whole spectrum can be used to classify cells using several clustering methods, such as principal-component analysis, principal-coordinate analysis, nonmetric multidimensional scaling, or T-distributed stochastic neighbor embedding. This information can also be used to construct dendrograms (25). Here, we used the preprocessed spectra to quantify the single-cell phenotypic diversity using Hill numbers. Every Raman peak corresponds to a different metabolite or a combination of metabolites, called components (*x*) (Fig. 1). To calculate the relative abundance of each peak, the intensity of the signal of each component was normalized by the sum of all intensities, and this information was then used in the Hill equations.

The order of diversity (*q*) can be any integer but is usually restrained to 0, 1, or 2, meaning that the richness and evenness of peaks can be weighed in the metric. Single-cell D_0_ (sc-D_0_) contains information about the number of components (*x*_i_) in the Raman spectra and is calculated as shown in equation 2 in Materials and Methods. sc-D_1_ informs about the evenness of each component and is described in equation 3. In this paper, we mostly focus on single-cell D_2_ (sc-D_2_) (*q *= 2), as it takes both richness and evenness of the Raman components into account.

### Sample size dependence of phenotypic diversity (sc-D_2_) measurements.

**FIG 2:**
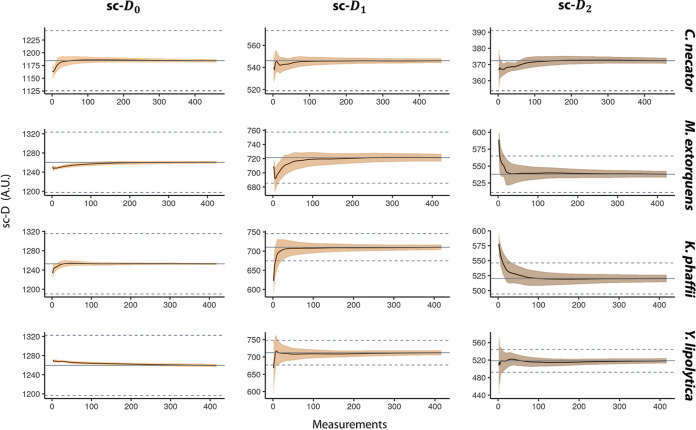
Effect of sampling size on the single-cell phenotypic diversity average. We calculated the average single-cell phenotypic diversity using the Hill equations (single-cell D_0_, D_1_, and D_2_) for an increasing number of measurements and repeated the calculation picking spectra randomly 1,000 times. We used the Raman spectra of four pure cultures and ∼450 measurements on each. The smears represent the standard deviations. The gray lines represent the average sc-D values of the total population, and the dashed lines represent a 5% deviation from the mean.

To understand the distribution of single-cell phenotypic diversity in a population, we did area scans in 2-μm droplets of four axenic cultures of Cupriavidus necator, Methylobacterium extorquens, Yarrowia lipolytica, and Komagataella phaffii, obtaining ∼450 measurements per culture. We calculated the average diversity estimation for an increasing number of spectra, bootstrapped 1,000 times. The average of the total number of measurements is plotted in gray in the graphs shown in Fig. 2, and 5% of this average is represented as a dotted gray line.

We looked at how many measurements were needed to calculate the population average (gray line) and how many are needed to have an accurate estimation (95%, dashed lines). For the estimation of sc-D_0_, few measurements (∼10 to 50) were needed to obtain the population average. The sc-D_1_ calculation grants a greater weight to high-intensity wavenumber and/or peaks of these components and required ∼100 measurements, although M. extorquens reaches it after ∼20 measurements. The sc-D_2_ estimation takes both the number of components and their evenness into account and needed between ∼50 (C. necator) to ∼180 (Y. lipolytica) measurements to estimate the population average.

### Case studies: phenotypic diversity quantification in stress-induced phenotypes.

**FIG 3:**
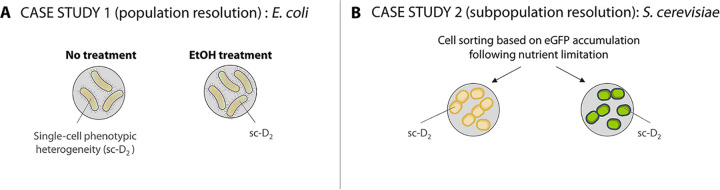
Overview of the case studies. (A) Study of two E. coli populations grown separately with ethanol in the medium or nontreated. (B) Two subpopulations were isolated from a S. cerevisiae culture based on the expression of the GFP-marked chimeric stress reporter after nutrient limitation. The Raman spectra of single cells were used to calculate their phenotypic diversity (sc-D_2_).

When stress is applied to a microorganism, a set of genes and proteins are expressed, changing the metabolic phenotype of the cell. This metabolic change can be captured by Raman spectroscopy, which collects information on the (bio)molecules present in individual cells. To compare stressed and nonstressed cells, we quantified their phenotypic diversity using our proposed methodology, as shown in Fig. 3. First, we compared two E. coli cultures growing under different conditions: with ethanol (stressed) or nontreated (control). Then, we compared two subpopulations of the same S. cerevisiae culture, separated based on their expression of the GFP stress reporter under nutrient-limiting conditions.

### Tracking E. coli population diversification dynamics following exposure to ethanol stress.

We used a data set from the study by Teng et al. (14) consisting of spectra of Escherichia coli sampled at different time points (5, 10, 20, 30, and 60 min, 3 h, and 5 h) after being cultured under standard conditions or with ethanol. There were three biological replicates of the cell culture, and 20 cells were measured per replicate.

**FIG 4:**
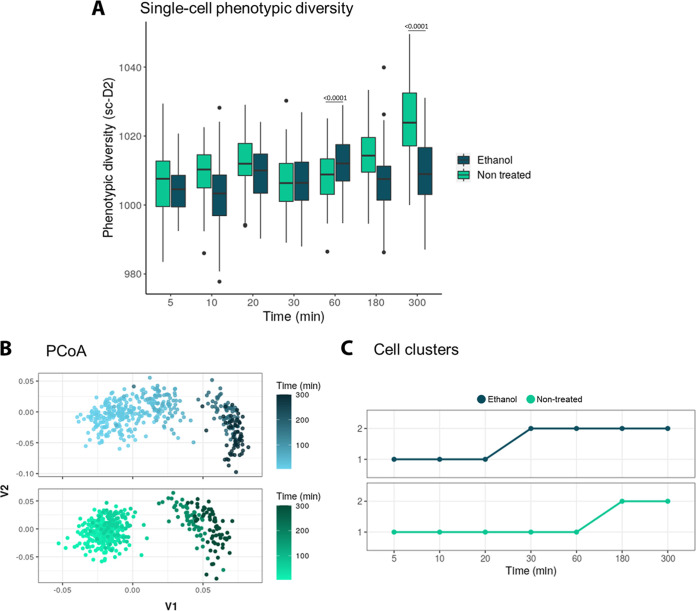
(A) Single-cell phenotypic diversity (sc-D_2_) of the stressed (ethanol treated) and nonstressed (nontreated) E. coli populations. Treatments and treatments over time were significantly different (two-way ANOVA, *P* < 0.0001). A *post hoc* Tuckey’s test showed that the ethanol and control groups are significantly different at time points of 60 min and 180 min (*P* < 0.0001). (B) Raman fingerprint of the stressed (ethanol treated) and nonstressed (nontreated) E. coli populations, plotted using principal-component analysis (PCoA). The time progression is represented with a darker color. Every point represents a single cell. (C) The clustering algorithm shows the phenotypic shift happens after 20 min for the ethanol-treated population and after 180 min for the control. Two phenotypes were found. Every point represents the average “phenotypic type” of the population. *N* = 60.

The stress-induced metabolic diversity of single cells was quantified using the sc-D_2_ Hill diversity metric, and the average diversity for each population (stress and nonstressed) was plotted (Fig. 4A). After testing for normality, a two-way analysis of variance (ANOVA) showed a significant difference between the ethanol and the control groups and also a significant difference in both the ethanol-treated and control groups over time (*P* < 0.0001). A *post hoc* Tukey’s test showed that the ethanol and control groups were significantly different at time points 60 min and 180 min (*P* < 0.0001). Then, we used a principal-coordinate analysis (PCoA), a common clustering method to visualize the dissimilarities in the fingerprints. The Raman fingerprints of the stressed and control cells were similar at the beginning and then shifted over time (Fig. 4B). We used a clustering algorithm to define exactly when this shift takes place: after 20 min for the ethanol-treated population and 180 min for the control population (Fig. 4C).

### Discriminating S. cerevisiae subpopulations following exposure to nutrient limitation.

An S. cerevisiae population was cultured under nutrient-limiting conditions. Based on GFP expression as an indicator of stress activation, we separated two subpopulations (one that activated the stress reporter and one that did not) using fluorescence-activated cell sorting (FACS). Then, we analyzed 65 cells in each subpopulation using Raman spectroscopy.

**FIG 5:**
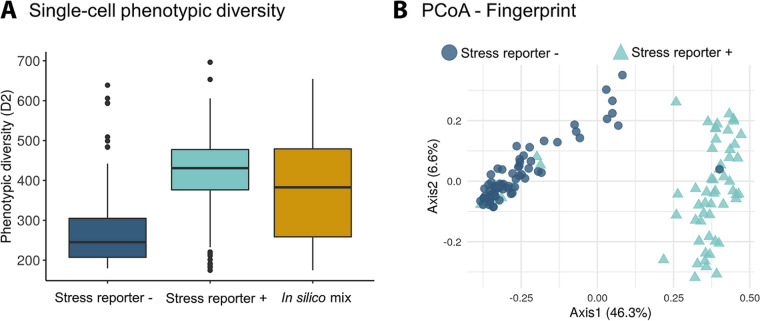
(A) Single-cell phenotypic diversity of a S. cerevisiae subpopulations with high or low stress reporter expression and an *in silico* mix of both groups. The *in silico* mix is a random selection of cells coming from the stressed and nonstressed populations. (B) Visualization of the stress-induced phenotypic change of Saccharomyces cerevisiae subpopulations with high or low stress reporter expression using principal-coordinate analysis (PCoA). Every dot is a single cell. The size of the dot corresponds to the single-cell phenotypic diversity (sc-D_2_). *N* = 65.

First, we calculated the single-cell phenotypic diversity (sc-D_2_) of the subpopulations with high (+) or low (−) stress reporter expression. To demonstrate that sc-D_2_ calculations were quantitative, we also created an *in silico* group by mixing the data of the two subpopulations (Fig. 5A). The *in silico* mix group was expected to have an average sc-D_2_. Then, we checked the dissimilarity of the fingerprints using PCoA (Fig. 5B). Two clusters were differentiated depending on the reporter expression.

**FIG 6:**
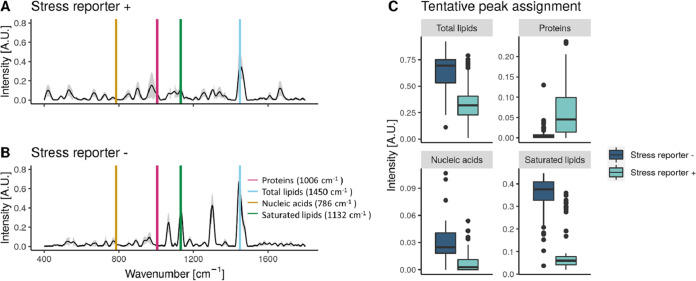
(A and B) Raman spectra of S. cerevisiae subpopulations with high (+) or low (−) expression of the stress reporter. The average of the spectra is plotted with a black line and the standard deviation in gray. The putative peaks corresponding to proteins, lipids, nucleic acids, and saturated lipids according to Teng and colleagues (14) are plotted over the spectra. (C) The intensity of the metabolic peaks highlighted in plots A and B for the subpopulations with high or low expression of the stress reporter. The peak assignment is tentative and needs to be validated by another technique. *N* = 65.

The information of the Raman spectra from each group was used to understand the effect of the stress reporter activation on the metabolic response of S. cerevisiae. Using a tentative assignment based on that by Teng et al. (14), we estimated the protein (1,006 cm^−1^), total lipid (1,450 cm^−1^), nucleic acid (786 cm^−1^), and saturated lipid (1,132 cm^−1^) contents in in the subpopulations with high or low stress reporter expression (Fig. 6). There can be spectral shifts between databases, due to the use of a different laser and instrument and/or because of the handling of the sample. To examine these phenomena, we took as a reference the 1,002-cm^−1^ peak, which corresponds to the aromatic amino acids phenylalanine and/or tyrosine. It is a very prominent band that is usually recognizable in biological samples (26). We observed that this peak occurred at 1,006 cm^−1^ in the S. cerevisiae data set, and so we accounted for a 4-cm^−1^ shift between data sets. We found that both groups have a significantly different metabolism: the subpopulation with high (+) expression of the stress reporter had a higher protein content but contained less total and saturated lipids and nucleic acids (Wilcoxon rank-sum test, *P* < 0.0001). However, this peak assignment is only tentative and needs to be validated using another technique. We cannot claim with certainty the exact molecular identity corresponding to each Raman wavenumber, as further explained in the Discussion section.

## DISCUSSION

This work shows how Raman spectra data can be used to study stress-driven metabolic heterogeneity at the single-cell level. The laboratory and computational workflow is relatively fast and nondestructive and can provide (semi)quantitative information about the biomolecular composition of cells. Although Raman spectroscopy has been previously used to detect stress-driven phenotypes (13), we argue that there is a need for quantitative single-cell measurements for phenotypic diversity and propose the use of Hill numbers. We chose Hill numbers for our calculations because they are widely used in microbial ecology.

To estimate the phenotypic diversity using the Hill diversity framework, we considered that each normalized Raman signal corresponds to a component (a single or multiple molecules) and that the intensity of these components is correlated with their quantity (27, 28) (Fig. 1). Although we chose to use the whole spectrum for this calculation, it is possible to select only the peaks. However, this could influence the resolution: algorithms for peak detection typically divide the spectrum according to a certain window size and look for the local maximum (29). Also, this algorithm did not take into account the width of components, which is a characteristic of the molecules. It would be interesting to study how these two phenomena—using only spectral peaks or using the width information—affect the single-cell diversity calculation. Additionally, some components with a close signal would be ignored, and the choice of window size would affect the final result. Additionally, the acquisition and preprocessing of the spectra can have an impact on the results. It would be worth exploring whether using a higher grating (i.e., 600 g/mm) could increase the resolution and result in a more accurate diversity calculation. Second, the region used for fingerprinting needs to be considered so that all the molecules relevant to address the hypothesis are reported. We used the 600- to 1,800-cm^−1^ region in this work, but it would be interesting to test the impact of using other spectral regions. When preprocessing, the methods chosen for baseline correction and normalization will have an impact on the intensity reported for the different components. The use of smoothing functions can lead to wrongfully assuming that certain spectral points are noise and thus needs to be considered carefully. Finally, aligning spectra when unnecessary can misplace the signals. Therefore, using the same preprocessing steps when comparing samples is crucial as well as detailing the preprocessing steps and providing the raw data.

To explore the importance of the sample size in these estimations, we used a large data set consisting of ∼450 Raman spectra from 2 axenic bacterial cultures (C. necator and M. extorquens) and 2 axenic yeast cultures (Y. lipolytica and *K. phaffii*). Then, the effect of the sampling size on the average single-cell phenotypic diversity and its standard deviation were calculated. Our results show that this is highly population dependent: for example, while C. necator only needed 15 spectra to approach the expected sc-D_2_ average, Y. lipolytica needed more than 150 measurements (Fig. 2). This could be due to a different degree of phenotypic diversity in the populations. Sample size should be explored for every experiment to make sure that the estimations are representative.

After developing the methodology to quantify single-cell phenotypic diversity, we applied it to two case studies to demonstrate its use. We focused on sc-D_2_, as it considers how many components are being expressed per cell and their evenness. In the first case study, we compared an ethanol-treated and a control E. coli population. We found that when E. coli is grown under standard conditions, there is a phenotypic shift after 60 min. This shift happens earlier in stressed cells (20 min) (Fig. 4C). The shift in the fingerprint in the control group could be due to the entering of log phase. Our group previously showed how E. coli cells start their log phase after ∼1 h of cultivation in rich medium and how, at different growth stages, bacteria change their phenotype (30). Although both the ethanol-treated and the control populations end up having similar phenotypes after 60 min, the stressed population has a lower metabolic diversity (Fig. 4A), a lower nucleic acid content, and higher protein and lipid contents. Clustering algorithms are useful to automatically identify phenotypes and quickly assess when the phenotype of a population has changed in a reproducible way. While here we use PCA, other metrics can be used, such as nonmetric multidimensional scaling (NMDS), *t*-distributed stochastic neighbor embedding (t-SNE) and other clustering methods. The choice of the clustering method should be based on the hypothesis and how important it is to conserve the distances between the cells and the relative size of the cluster.

In the second case study, we analyzed the response of two S. cerevisiae subpopulations. When under nutrient-limiting conditions, S. cerevisiae resorts to a bet-hedging strategy where some yeasts will enter a quiescent state and others will activate a stress-induced response (31). The strain used in this experiment produces GFP upon activation of nutritional stress, so when the S. cerevisiae culture diversified into two populations—with either high or low expression of the stress reporter—these were separated using FACS and analyzed with Raman spectroscopy. Because the Raman spectroscope used has a 785-nm laser, we do not expect the fluorescent signal (excited at 510 nm) to be picked up with this instrument. Single-cell phenotypic diversity (sc-D_2_) in the stressed subpopulation is higher than in the nonstressed population (Fig. 5A). As expected, the *in silico* mix shows a diversity that is close to the average for both subpopulations. We then checked that the subpopulations with high and low stress reporter expression had a different fingerprint using PCoA, a tool widely used for Raman spectra in microbial ecology. This confirmed that the fingerprints of both subpopulations are visibly different (Fig. 5B). Using the metabolic information contained in the Raman spectra, we found a higher nucleic acid content in the nonstressed subpopulation (in line with the findings of Teng et al. [14] in stressed E. coli cells). This could be explained by the higher ribosome content in nonstressed cells. We also found that the stress response triggered by the activation of the chimeric promoter results in a raise in protein production (Fig. 6), similar to the results found in stressed E. coli cells. We cannot, on the other hand, exclude that GFP production may somewhat influence the molecular fingerprint of cells (e.g., via depletion of amino acid pools, reducing ribosomal availability). Also, differences in protein abundance between the stressed and nonstressed subpopulations could be (at least partially) due to the GFP protein itself. To explore these possibilities, a proteomics and/or transcriptomics analysis at the single-cell level would be required. The choice of this promoter based on a fusion of *glc3* and *hsp26* as a single proxy to define a metabolically stressed population is cross validated by these findings, which show two clearly metabolically distinct subpopulations. It is important to mention that these metabolic estimations were made using an external database and should be considered tentative assignments. To confirm these results, a second technique should be used. Other authors that previously explored stress-induced responses in yeast using Raman spectroscopy found that the 1,602-cm^−1^ band, which corresponds mainly to ergosterol production (32), can indirectly measure oxidative stress and cellular metabolism after atmospheric or nutrient changes (33). This band can be used as a label-free *in vivo* activity indicator in both S. cerevisiae and Schizosaccharomyces pombe.

Finally, we explored whether the number of cells measured in both case studies was enough to capture the diversity of the cultures. In S. cerevisiae, 65 cells were enough to estimate single-cell diversity and most biomolecules (see Fig. S2 and S3 in the supplemental material). However, to properly estimate the protein content in the nonstressed subpopulation, more cells would have been needed. The laser spot used for these measurements had a diameter of 1.7 μm; thus, the spectra of the yeasts could have varied depending on the position of the laser inside the yeast. To avoid this variation, the operator aimed at the center of the yeast to the best of her ability. The center of S. cerevisiae most likely contains nuclear components, although the nucleus is not necessarily located at the center of cells, and its shape can deviate consistently from that of a sphere (34). This deviation occurs most notably under conditions of starvation, hence, very similar to those used to induce GFP expression in our study. Apart from nuclear information, we expect the spectra to bear information over the nuclear surroundings (most notably the cytosol), and the biomolecules positioned on the same longitudinal plane as the nucleus. Additionally, Fig. S3 shows how after measuring 40 to 50 cells, the measurements become quite representative of the population. However, a space-resolved experiment would help in gaining insight into the effect of stress in the cell wall or other structures of S. cerevisiae. In the E. coli population, we tested the sample size in the ethanol-treated population at time points of 5 min and 300 min. Very few cells are needed to have a representative single-cell diversity estimation: the sc-D_0_ is the same for all cells (Fig. S4). This metric looks at the number of components present in each cell, which in this case, seem to be the same for all individuals. It could be that these cells express the same molecules but different amounts and/or an artifact of the preprocessing carried out by Teng et al. (14), which could have erased some of the smaller peaks. This highlights the importance of making the raw data available, following the trends of other disciplines such as new-generation sequencing (NGS) or flow cytometry.

Inferring metabolic expression from Raman spectra in microbial cells is not without challenges. For instance, many databases propose different peaks to identify the same biomolecules. In the manuscript, we have chosen those presented in the study by Teng et al. (14) to be able to compare the results they found in E. coli with those we found in S. cerevisiae. To account for the wavelength shift between these two databases (due to the use of a different laser and instrument and/or because of the handling of the sample), we took as a reference the 1,002-cm^−1^ peak, which corresponds to the aromatic amino acids phenylalanine and/or tyrosine. It is a very prominent band that is usually recognizable in biological samples (26). This is at 1,006 cm^−1^ in the S. cerevisiae data set, and so we accounted for a 4-cm^−1^ shift between data sets. When studying the content of biomolecules with Raman spectroscopy, we also need to consider that some molecules are not Raman active (i.e., their chemical bonds have a weak signal) and thus will not be reflected in the spectra. Conversely, some Raman active molecules can be overrepresented in the analysis because certain chemical bonds exhibit a strong Raman signal. Also, Raman peaks can correspond to several molecules due to the presence of shared chemical bonds. These limitations should be considered when using Raman spectroscopy for microbial ecology. A better assignment of the Raman signals will also contribute to an improved understanding of the metabolic changes driving single-cell phenotypic heterogeneity.

Most ecological studies use low-dimensional physiological data or use single-marker gene expression to understand microbial populations. Raman spectroscopy is a promising single-cell technology able to quantify phenotypic diversity in individual cells, identify changes in phenotypes, and infer metabolic information (semi)quantitatively. This tool will allow microbial ecologists to go beyond community measurements and shed light on how heterogeneity shapes communities.

### Conclusions.

- Raman spectrum data can be used to quantify single-cell stress-driven phenotypic diversity in microbial populations.
- Raman spectral points correspond to different chemical bonds (or to multiple ones), which are expressed with a certain intensity and evenness. This information can be combined with the Hill diversity framework to estimate the phenotypic diversity of single cells. We show that these methods can be used to study changes at the population and subpopulation levels in various microbial systems.
- We propose an automatic classification of phenotypic groups using clustering methods. As Raman spectroscopy can detect stressed phenotypes, we propose it as a tool for monitoring microbial populations in bioproduction.

## MATERIALS AND METHODS

### Data sets.

The strains used and the incubation medium are described in Table 1. We performed ∼450 measurements in 4 axenic cultures using Raman spectroscopy. Samples were cultured at 28°C with 120-rpm orbital shaking. Each strain was recultivated via transferring 10% (vol/vol) of active culture in fresh liquid medium (described in Table 1) every 24 to 48 h for 2 months. Cultures were harvested by centrifugation at 6,603 × *g* for 5 min, washed with 0.1 M phosphate-bufferd saline (PBS), and stored at −4°C until further use.

**TABLE 1:** List of organisms and media used to grow them

Organism	Liquid medium
Cupriavidus necator LMG 1199	Nutrient broth (Oxoid CM0001)
Methylobacterium extorquens DSM 1338	Nutrient broth with 1% methanol
Yarrowia lipolytica ATCC 20362	YM broth (BD 271120)
*Komagataella phaffii* ATCC 76273	Sabouraud broth (BD 238230)

### Case studies: single-cell phenotypic diversity quantification in stress-induced phenotypes.

To test the capacity of the single-cell phenotypic diversity (sc-D_2_) calculation to identify metabolic changes, we used two case studies. First, we studied two E. coli populations that had been grown together under different conditions: one was treated with ethanol while the other was not. Second, an S. cerevisiae culture was grown under nutrient-limiting conditions, which resulted in differential expression of the chimeric stress reporter (tagged with enhanced GFP [eGFP]). The two subpopulations (high-expressing and low-expressing eGFP) were isolated (Fig. 3).

### Population resolution: E. coli exposed to ethanol.

The data set from Teng et al. (14) was used to validate the diversity calculations. According to their manuscript, this data set consists of Raman spectra of Escherichia coli at different time intervals (5, 10, 20, 30, and 60 min, 3 h, and 5 h) after being cultured with different chemical stressors. We used the ethanol-treated samples and the controls to illustrate our point. The data set consists of three biological replicates of the cell culture, and we measured 20 cells per replicate.

### Subpopulation resolution: S. cerevisiae after nutrient limitation.

The prototrophic haploid yeast strain Saccharomyces cerevisiae CENPK 113-7D was used in this study (35). eGFP was produced under the control of a chimeric promoter composed of fragments of the *HSP26* and *GLC3* promoters. The promoter sequence was previously published (chimera 2 in [36]). A synthetic construct containing the promoter, the eGFP gene, and the G418 resistance marker was integrated in the genome via homologous recombination at the *uga1* site. The correct insertion was confirmed via PCR analysis and lack of growth on gamma-aminobutyrate (GABA) as the sole nitrogen source.

Samples were collected after 10 residence times in a continuous culture operated at D = 0.1 h^−1^ in a 2-liter stirred-tank bioreactor with 1-liter operating volume. Defined yeast mineral medium containing 7.5 g liter^−1^ was used (37). The culture temperature was maintained at 30°C, the stirrer speed at 1,000 rpm, and the air provision at 1 volume of air per volume of culture per min (vvm). The culture pH was controlled at 5.0 through the automated addition of either 25% KOH or 25% H_3_PO_4_.

Before cell sorting, samples were fixed in formaldehyde 4% according to the protocol from García-Timermans (25). Paraformaldehyde is known to preserve the Raman spectral features better than other fixatives, such as ethanol or glutaraldehyde (38). Upon reaching steady state in nutrient-limited continuous culture, we collected a cell suspension from the bioreactor and diluted it 10 times in PBS (Thermo Fischer Scientific, Belgium) and further analyzed and sorted it using a FACSaria (Becton, Dickinson, Belgium). Two distinct subpopulations were sorted using fluorescence-activated cell sorting (FACS): the first subpopulation exhibited a high GFP content (high GFP) and the second a low GFP content (low GFP). Cells were collected following an enrichment sorting mode, in fractions containing 10^6^ cells of each subpopulation. Gating details used for cell sorting can be found in Supplemental Fig. S1.

10.1128/mSphere.00806-20.1 FIG S1 (A) FACS gating strategy to separate high- and low-GFP-expressing subpopulations in an S. cerevisiae culture following nutrient limitation. The GFP marker is on a chimeric stress reporter. (B) Purity of the high GFP and low GFP separated fractions. Download FIG S1, TIF file, 0.4 MB. Copyright © 2020 García-Timermans et al. 2020 García-Timermans et al. This content is distributed under the terms of the Creative Commons Attribution 4.0 International license.

10.1128/mSphere.00806-20.2 FIG S2 Effect of sampling size on the single-cell phenotypic diversity average of S. cerevisiae. We calculated the average single-cell phenotypic diversity using the Hill equations (single-cell D_0_, D_1_, and D_2_) for an increasing number of cells in two S. cerevisiae subpopulations, with either high or low stress reporter expression. We repeated the calculation, picking cells randomly 1,000 times. The smears represent the standard deviations. The gray lines represent the average sc-D values of the total population, and the dashed lines represent a 5% deviation from the mean. *N* = 65. Download FIG S2, TIF file, 0.5 MB. Copyright © 2020 García-Timermans et al. 2020 García-Timermans et al. This content is distributed under the terms of the Creative Commons Attribution 4.0 International license.

10.1128/mSphere.00806-20.3 FIG S3 Effect of sampling size on the estimation of biomolecules in S. cerevisiae. We calculated the average of total lipids (1,450 cm^−1^), saturated lipids (1,132 cm^−1^), proteins (1,006 cm^−1^), and nucleic acids (786 cm^−1^) in two S. cerevisiae subpopulations two S. cerevisiae subpopulations, with either high or low stress reporter expression for an increasing number of cells. We repeated the calculation, picking cells randomly 1,000 times. The smears represent the standard deviations. The gray lines represent the average sc-D values of the total population, and the dashed lines represent a 5% deviation from the mean. *N* = 65. Download FIG S3, TIF file, 0.3 MB. Copyright © 2020 García-Timermans et al. 2020 García-Timermans et al. This content is distributed under the terms of the Creative Commons Attribution 4.0 International license.

10.1128/mSphere.00806-20.4 FIG S4 Effect of sampling size on the single-cell phenotypic diversity average of E. coli. We calculated the average single-cell phenotypic diversity in an E. coli population after being exposed to ethanol for 5 and 300 min. We used the Hill equations (single-cell D_0_, D_1_, and D_2_) for an increasing number of cells and repeated the calculation, picking cells (*n* = 60 cells) randomly 1,000 times. The smears represent the standard deviations. The gray lines represent the average sc-D values of the total population, and the dashed lines represent a 5% deviation from the mean. Download FIG S4, TIF file, 0.6 MB. Copyright © 2020 García-Timermans et al. 2020 García-Timermans et al. This content is distributed under the terms of the Creative Commons Attribution 4.0 International license.

### Raman spectroscopy.

For the S. cerevisiae samples, three drops of 2 μl were placed on a CaF_2_ slide (11-mm-diameter by 0.5-mm polished disc; Crystran Ltd.). We measured 65 single cells using a WITec Alpha300R+ with a 785-nm excitation diode laser (Topotica) and a 100×/0.9 numerical aperture (NA) objective (Nikon) with 40 s of exposure and 1 accumulation using a 300-g/mm grating. The laser was positioned at the center of the yeast to the best of the operator’s ability.

For the samples from C. necator, M. extorquens, Y. lipolytica, and *K. phaffii*, we used a sterile tip to smear a 2-μl drop and obtain a more uniform surface. Then, using the area scan function, ∼450 points were measured using 5 s of exposure and 1 accumulation with a 300-g/mm grating. Area scanning allows us to rapidly obtain a high number of measurements. However, because we did not individually aim for single cells using the microscope, we cannot claim these are single-cell measurements and, hence, refer to these spectra as “points.”

As a control for the instrument performance, a silica gel slide was measured with a grating of 300 g/mm, with a 1-s time exposure and 10 accumulations. Laser power was monitored to detect possible variations. More information can be found in the Raman metadata aid (see Table S1) collected following the guidelines of García-Timermans et al. (25).

10.1128/mSphere.00806-20.5 TABLE S1 Metadata aid for Raman spectra. Download Table S1, DOCX file, 0.1 MB. Copyright © 2020 García-Timermans et al. 2020 García-Timermans et al. This content is distributed under the terms of the Creative Commons Attribution 4.0 International license.

### Data analysis.

The data analysis was conducted using R (R version 3.6.2) in RStudio version 1.2.1335 (39, 40). Figures were produced using the package ggplot2 (version 3.3.2) and ggpubr (version 0.2) (41, 42).

### Preprocessing.

We manually eliminated the spectra that contained cosmic rays. The remaining spectra were preprocessed using the R packages “MALDIquant” (version 1.16.2) (29) or “HyperSpec” (version 0.99.20200527) (43). To reduce the noise in the spectra, we smoothed it using the spc.loess() function. The 400 -to 1,800-cm^−1^ region of the spectrum, which contains the biological information in bacteria, was selected for fingerprinting. The baseline was corrected for instrumental fluctuations and background noise using the sensitive nonlinear iterative peak (SNIP) algorithm (using 10 iterations). Then, the spectra were normalized using the calibrateIntensity() function with the method total ion current (TIC) and aligned per group with the alignedSpectra() function. The HyperSpec object was smoothed using the spc.loess() function. These preprocessed data were used to calculate the single-cell phenotypic diversity and principal-coordinate analysis.

### Single-cell phenotypic diversity calculation for single cells with Raman spectroscopy.

The Hill equations were adapted in the manuscript to quantify the phenotypic diversity of single cells (sc-D_2_) using preprocessed Raman spectra. Every Raman signal corresponds to single or multiple metabolites, which we have called components (*x*). The relative abundance of each component was normalized by calculating their relative abundance. Then, they were used in the Hill equation as described in Results.

Hill numbers are commonly used to calculate microbial diversity based on 16S rRNA gene sequencing techniques but have also been applied to flow cytometry, yielding similar results (17, 44). Although there are many diversity calculations, Hill numbers are widely used. They are also known as the effective number of species, as they express in intuitive units the number of equally abundant species that are needed to give the same value of the diversity measure. Hill numbers respect other important ecological principles, such as the replication principle, which states that in a group with *N* equally diverse groups that have no species in common, the diversity of the pooled groups must be *N* times the diversity of a single group. The general Hill equation is:

(1) $$D={\left(\sum p_{i}^{q}\right)}^{1/\left(1-q\right)}$$

where *p_i_* is the relative abundance of an *i* number of taxa, and *q* is the sensitivity parameter, also known as the diversity order, which can be 0, 1, or 2. The diversity index of order 0 (D_0_, when *q* = 0) corresponds to the taxon richness (the total number of species in the sample), D_1_ weighs each taxon proportionally to their abundance, and D_2_ considers both richness and evenness. When *q* = 1, the result is undefined, but Hill (45) proved its limit to be

(2) $$D_{1}=\exp\left(-\sum p_{i}\text{ ln }p_{i}\right)$$

When *q* = 2, the Hill equation corresponds to

(3) $$D_{2}=\frac{1}{\sum p_{i}{}^{2}}$$

More information on the diversity measures used in microbial ecology and the advantages of Hill numbers can be found in reports by Chao et al. (15) and Daly et al. (16).

### Statistical analysis.

The significance of the stress-induced metabolic diversity (sc-D_2_) of E. coli cells was evaluated. First, we tested the normality of the groups using ggdensity() and ggqqplot() from the package “ggpubr” (version 0.2). The significance between the treatment and control groups was tested using ANOVA with the function aov(), and *post hoc* testing was done using Tukey_HSD(), both functions from the package “stats” (version 3.6.3).

We also evaluated the statistical difference in the expression of total lipids, proteins, nucleic acids, and saturated lipids between the stressed and control S. cerevisiae subpopulations. After testing the normality of the groups using ggdensity() and ggqqplot() from the package “ggpubr” (version 0.2), we used the Wilcoxon test with the function wilcox.test() from the package “stats” (version 3.6.3).

### Principal-coordinate analysis.

We performed a principal-coordinate analysis (PCoA) on the processed spectra using the custom function beta.div.Raman(). This function first normalizes each spectral point by dividing it by the maximum value for that wavelength in the data set. Then, it calculates the dissimilarity of the spectra using Bray-Curtis through the vegdist() function of the “vegan” package (version 2.5.6). Finally, the principal-coordinate analysis is calculated using the cmdscale function of the “stats” package (version 3.6.3).

### Sampling size.

We measured of four axenic cultures of C. necator, M. extorquens, Y. lipolytica, and *K. phaffii.* First, we put a 2-μl droplet of the sample on a slide. Once it was dry, we did and area scan, obtaining ∼450 measurements per culture. Then, we calculated the phenotypic diversity of single cells (sc-D_2_). We did 1,000 simulations where we calculated the average D_2_ when using an increasing number of bootstrapped spectra. The average and standard deviation of these 1,000 simulations were plotted.

### Subpopulation types.

Subpopulation types were calculated by adapting the code for flow cytometry data. The method was originally intended to separate sample clusters, while in its application for Raman spectroscopy, we aimed to identify and differentiate cell clusters (16).

First a PCA is performed to reduce the dimensionality of the data. A reduced data set with the principal components that explain the majority of the variance (>40%) is used to calculate the optimal number of clusters using the silhouette index and then used partitioning around medoids (PAM) as a clustering algorithm to determine to which cluster cells belong to. This was conducted using the pam() function from the package “cluster” (version 2.1.0). Once every cell was assigned a phenotype (cluster), the median phenotype to which the (sub)population corresponds was calculated.

### Data availability.

The analysis pipeline, raw data, and code to reproduce the analysis shown in the manuscript can be found in the repository at https://github.com/CMET-UGent/Raman_PhenoDiv. The data set from the study by Teng et al. (14) was used to validate the diversity calculations as well as the “subpopulation type” definition.
