# Supplementary figures and images for: Raman Spectroscopy-Based Measurements of Single-Cell Phenotypic Diversity in Microbial Populations
Source: mSphere. 2020 Oct 28;5(5):e00806-20. doi: 10.1128/mSphere.00806-20 (PMC7593600; doi:10.1128/mSphere.00806-20)

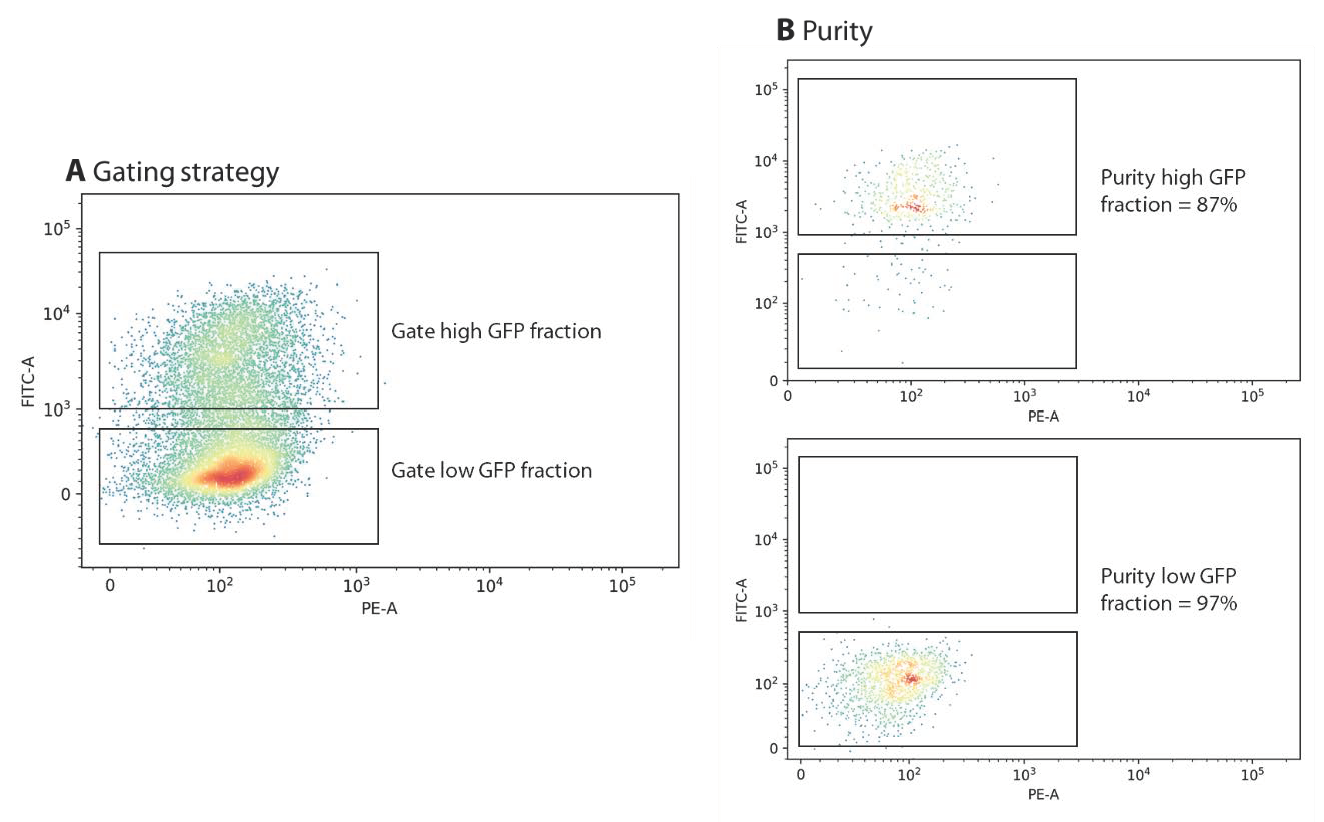

Supplement: FIG S1 [file mSphere.00806-20-sf001.tif]

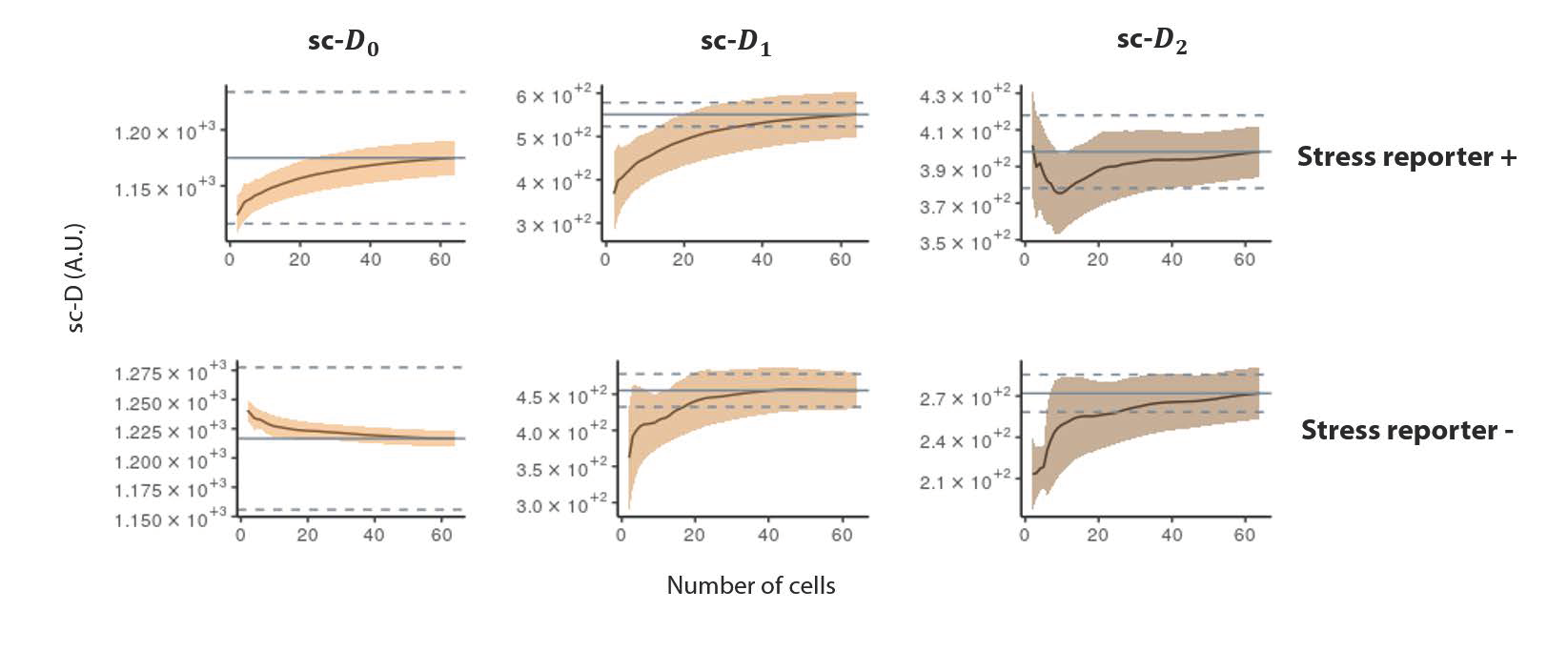

Supplement: FIG S2 [file mSphere.00806-20-sf002.tif]

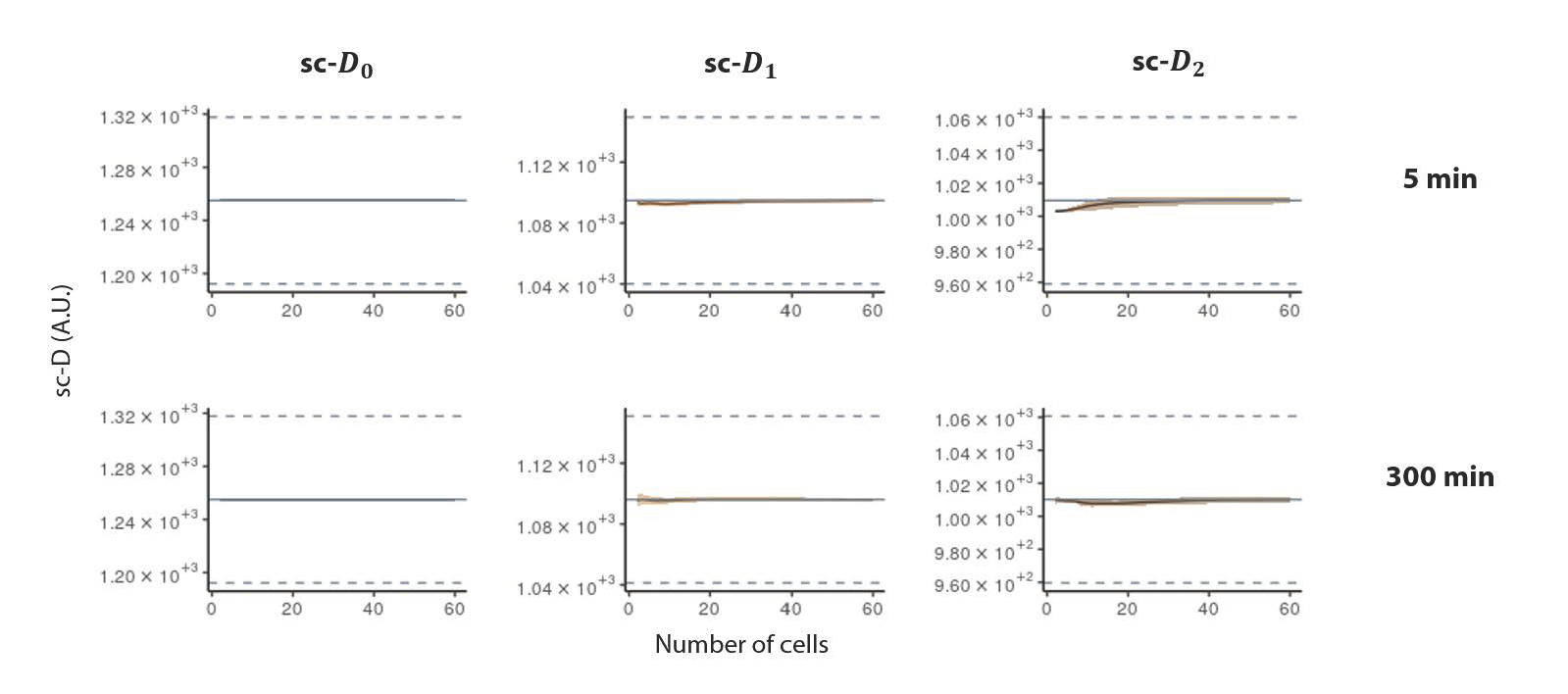

Supplement: FIG S3 [file mSphere.00806-20-sf003.tif]

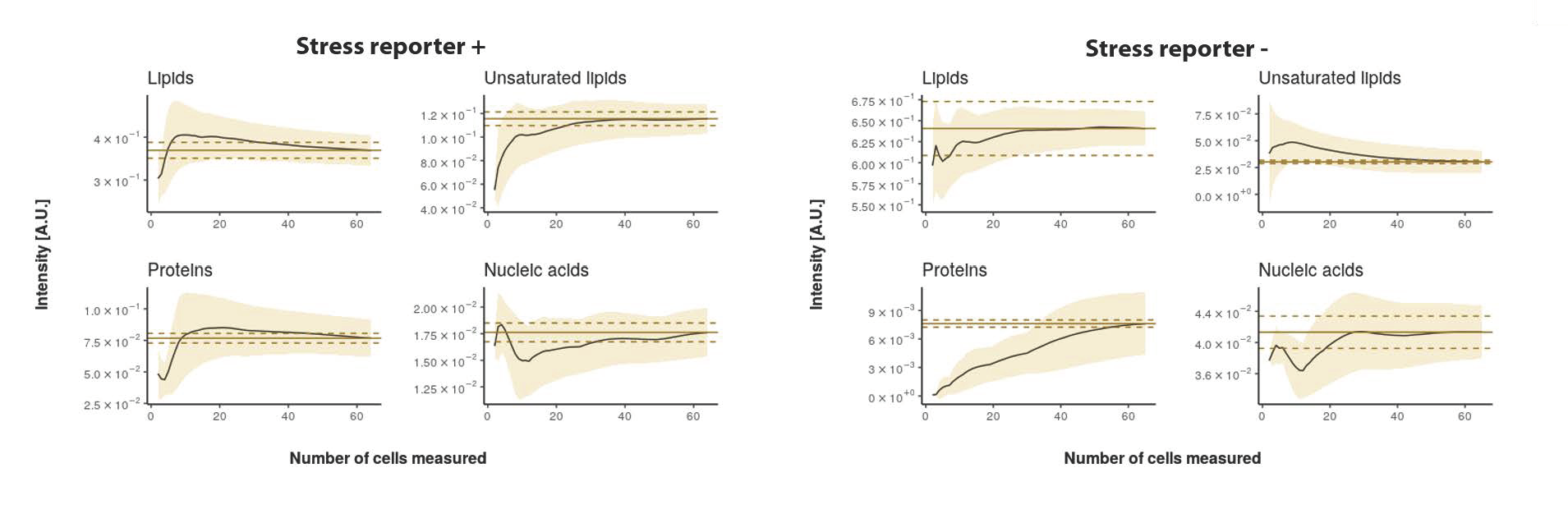

Supplement: FIG S4 [file mSphere.00806-20-sf004.tif]
