# Supplementary material for: Raman Spectroscopy-Based Measurements of Single-Cell Phenotypic Diversity in Microbial Populations
Source: mSphere. 2020 Oct 28;5(5):e00806-20. doi: 10.1128/mSphere.00806-20 (PMC7593600; doi:10.1128/mSphere.00806-20)
Supplement: TABLE S1 [file mSphere.00806-20-st001.docx]

| **Experiment overview:** | |
| --- | --- |
| Hypothesis | The *Saccharomyces cerevisiae* strain CENPK 113-7D with an eGFP tag in its chimeric promoter was used in this experiment. After growing the cells, they were fixed in formaldehyde 4% and sorted into two groups depending on their GFP expression (high or low) using fluorescence-activated cell sorting (FACS). Then 65 cells of each group were measured using Raman spectroscopy.  To understand the number of measurements that need to be made by sample, we did ~450 measurements in 4 axenic cultures of *C. necator, M. extorquens, Y. lipolytica* and *K. phaffi* |
| Variable(s) tested | Subpopulation differences |
| Conclusions | Our pipeline can discriminate the two subpopulations |
| Quality control (internal/external) | Silica gel check |
| **Samples and sample acquisition** | |
| Material and source | *Saccharomyces cerevisiae*  *C. necator, M. extorquens, Y. lipolytica* and *K. phaffi* |
| Growing conditions/sampling | See description in materials and methods |
| Filename format: <Sample>_<Measurement> | |
| Label in the samples | N/A |
| Fixation method | formaldehyde 4% |
| Integration time | 40 s |
| Accumulations | 1 |
| Grid | 300 –mm/g |
| **Instrument** | |
| Laser | 785 nm excitation diode laser (Toptica). 130 mW of power before the objective. |
| Quality control | For the *S. cerevisiae* samples, a silica gel sample was measured with a grating of 300 –mm/g, with a 1 second time exposure and 10 accumulations.  For the samples from *C. necator, M. extorquens, Y. lipolytica* and *K. phaffi,* ~450 points were measured using 5 sec of exposure and 1 accumulation with a 300 -mm/g grating.  Laser power was also monitored to detect possible variations. |
| Objective used (magnification ) / Numeric aperture (NA) | 100x/0.9 NA (Nikon) |
| Camera | -70 °C cooled CCD camera (iDus 401 BR-DD, ANDOR) |
| Dry/water/oil objective | Dried samples |
| Model of spectroscope | WITec Alpha300R+ |
| Other specifications (chromatic/flat field correction/other) |  |
| **Data analysis** | |
| Background subtraction method (if used) | No. Measurements with cosmic rays were deleted |
| Normalization method (peak /min-max /area under-curve /other) | Area under the curve (‘Total Ion Count’) |
| Smoothing and interpolation (if done) | Smoothing, baseline correction, normalization and alignment (per group) |
| Statistics/Machine learning algorithm | Wilcoxon test for pairwise comparisons between two groups. |
| Accessibility | https://github.com/CMET-UGent/Raman_PhenoDiv |
| Other relevant information |  |
